# Emerging Insights on the Biological Impact of Extracellular Vesicle-Associated ncRNAs in Multiple Myeloma

**DOI:** 10.3390/ncrna6030030

**Published:** 2020-08-05

**Authors:** Stefania Raimondo, Ornella Urzì, Alice Conigliaro, Lavinia Raimondi, Nicola Amodio, Riccardo Alessandro

**Affiliations:** 1 Department of Biomedicine, Neurosciences and Advanced Diagnostics (Bi.N.D), Section of Biology and Genetics, University of Palermo, 90133 Palermo, Italy; ornella.urzi@unipa.it (O.U.); alice.conigliaro@unipa.it (A.C.); 2 IRCSS Istituto Ortopedico Rizzoli, SC Scienze e Tecnologie Chirurgiche–SS Piattaforma Scienze Omiche per Ortopedia Personalizzata, 40136 Bologna, Italy; lavinia.raimondi@ior.it; 3 Department of Experimental and Clinical Medicine, Magna Graecia University of Catanzaro, 88100 Catanzaro, Italy; amodio@unicz.it; 4 Institute for Biomedical Research and Innovation (IRIB), National Research Council (CNR), 90146 Palermo, Italy

**Keywords:** extracellular vesicles, non-coding RNA, multiple myeloma, progression, drug resistance, biomarkers

## Abstract

Increasing evidence indicates that extracellular vesicles (EVs) released from both tumor cells and the cells of the bone marrow microenvironment contribute to the pathobiology of multiple myeloma (MM). Recent studies on the mechanisms by which EVs exert their biological activity have indicated that the non-coding RNA (ncRNA) cargo is key in mediating their effect on MM development and progression. In this review, we will first discuss the role of EV-associated ncRNAs in different aspects of MM pathobiology, including proliferation, angiogenesis, bone disease development, and drug resistance. Finally, since ncRNAs carried by MM vesicles have also emerged as a promising tool for early diagnosis and therapy response prediction, we will report evidence of their potential use as clinical biomarkers.

## 1. Introduction

Multiple myeloma (MM) is a hematological disease caused by the monoclonal expansion of malignant plasma cells (PCs) within the bone marrow. It evolves from two asymptomatic conditions, namely monoclonal gammopathy of uncertain significance (MGUS) and multiple myeloma “smoldering” (SMM) [1]. Diagnostic criteria for the different stages include a percentage of infiltration of bone marrow PCs of less than 10% in MGUS that increases to up to 60% in subjects with SMM who have a higher risk of progression to overt disease [2,3]. The major complication observed in patients with MM is the occurrence of osteolytic lesions in the later stages of the neoplasm.

The therapeutic scenario of MM has evolved in the last two decades; currently, the combination of proteasome inhibitors such as bortezomib with immunomodulatory agents like lenalidomide and pomalidomide is the preferred pharmacological choice [4]. The use of bisphosphonates is intended for the management of patients with osteolytic bone disease [5,6]. However, MM, unfortunately, remains an incurable disease associated with a high mortality rate, due to late diagnosis [7,8] or resistance to pharmacological treatments [9,10].

Therefore, unraveling the mediators and molecular mechanisms involved in disease pathogenesis is mandatory to identify new therapeutic targets; at the same time, the development of non-invasive tools for early and differential diagnosis and monitoring of the therapeutic response are needed.

To date, growing evidence reveals how the multi-directional and dynamic interactions between MM cells and other components of the bone marrow microenvironment play a critical role in several steps of disease occurrence and progression. The tumor microenvironment consists of an extracellular matrix and cellular elements such as endothelial cells (ECs) [11,12], mesenchymal stem cells (MSCs) [13,14], osteoblasts (OBs) [15,16], osteoclasts (OCs) [17,18], and immune cells [19,20,21]. All these cell types are influenced by MM cells through cell–cell interactions or the release of secreted factors, which in turn contribute to tumor growth and survival.

Extracellular vesicles (EVs) are among the leading participants encountered in this crosstalk. They are a population of lipoproteic structures, heterogeneous in size and content, released in the extracellular space by all cell types [22,23]. EVs were initially recognized as a cellular route through which cells could discard undesired components [24]; today, in-depth investigations into their biogenesis and their content have indicated their implication in different physiological and pathological processes.

Overall, most of the studies investigating the role of EVs as possible mediators of intercellular communication have demonstrated that these vesicles contain nucleic acids, like messenger RNA (mRNA) [25], non-coding RNAs (ncRNAs), and DNA [26], which could be delivered to target cells, thus influencing their phenotype [25]. In parallel, the presence of EVs in various biological fluids and whose content reflects that of the cells of origin makes EVs valuable diagnostic and prognostic tools.

The RNA cargo of EVs is heterogeneous and differs between different biological fluids [27], among vesicles of different cell types, and even within vesicles released from the same cell type [28]. In this regard, ncRNAs have emerged as being among the most abundant nucleic acids in vesicles [29,30].

The main purpose of this review is to report and discuss those studies aimed at disclosing the role of EVs in the pathobiology of MM and to highlight EVs’ diagnostic and prognostic potential. In this context, priority has been given to the discussion of the works correlating the biological effects of EVs to their ncRNA cargo.

## 2. Non-Coding RNAs in Extracellular Vesicles

The family of ncRNA includes all the ribonucleic acids that will not be translated into proteins. Some of them, the housekeeping ncRNAs, are constitutively expressed and participate in the translation process, i.e., the transfer RNAs (tRNAs) and the ribosomal RNAs (rRNAs), or in splicing as the small nuclear RNAs (snRNAs). However, the broadest category includes inducible ncRNAs that anneal complementary sequences in DNAs or RNAs, thus largely controlling gene expression, e.g., Piwi RNA, microRNA, and long non-coding RNAs.

Basing on their positions, ncRNAs are defined as intragenic or intergenic, but more often, they are classified based on their size as small non-coding RNAs and long non-coding RNAs (lncRNAs), the latter with sizes longer than 200 nucleotides. Several studies demonstrate that plasma cell dyscrasias are regulated by different classes of non-coding RNAs [31,32].

MicroRNAs (miRNAs) are a class of single-stranded, endogenous, small non-coding RNAs containing around 20–25 nucleotides implicated in various cellular pathways. These are transcribed as pri-miRNAs by RNA polymerase II or III or processed from longer non-coding RNA or the introns of protein-coding genes (miRtrons) [33]. Their action on the control of gene expression takes place at the cytoplasmic level, where, once reached, the mature form will be recruited by the ARGONAUTE (AGO) proteins, together with the RNA-induced silencing complex (RISC), to regulate gene expression at the post-transcriptional level through partial complementarity to target mRNAs. miRNAs are involved in complex regulatory circuits linked to biological processes and endowed with prognostic significance in cancers including MM [34].

Piwi-interacting RNAs (piRNAs) work similarly; these are highly enriched in the germline tissues where, in combination with Piwi proteins, they selectively silence mobile genetic elements (transposons).

LncRNAs localize both in the nucleus and in the cytoplasm of cells, thus controlling gene expression at different levels. Inside the nucleus, lncRNAs can control chromatin condensation, such as the X-inactive-specific transcript (*XIST*) that has a role in the formation of Barr bodies reviewed in [35]. However, the ability to recruit chromatin modifiers as the polycomb complex is common to several lncRNA, i.e., *HOTAIR* (Hox antisense intergenic RNA), antisense non-coding RNA in the INK4 locus (*ANRIL*), and metastasis-associated lung adenocarcinoma transcript 1 (*MALAT1*) [36]. In addition, by physical interaction with DNA, lncRNAs regulate transcription by favoring or inhibiting transcription factor recruitment and RNA polymerase activity [37,38]. Meanwhile, by orchestrating protein and nucleic acid interaction, lncRNAs give shape to the nuclear bodies [39]. Matured lncRNAs, provided with 5′-capping, polyadenylation, and methylation of adenosine (N6-methyladenosine, m6A), are exported to the cytoplasm, where they control post-transcriptional gene regulation. Once associated with RNA binding proteins (RBPs) such as HuR, they compete with mRNAs, thus controlling its stability or alternative splicing [40]. Recent work aimed at correlating lncRNA expression levels with the onset/progression of malignancies has also disclosed the capability of lncRNAs to sponge miRNAs, thus protecting mRNAs from miRNA-mediated targeting; this was largely demonstrated, e.g., for *lncH19*, *NEAT1*, *MALAT1*, and *HOTAIR* [41,42,43,44,45,46].

Strongly involved in the regulation of mRNA stability is the family of circular (circRNAs), a family of ncRNAs lacking the 5′ or 3′ ends, generated from the splicing machinery through a process called back-splicing. circRNAs emerged relatively recently and were found to be involved in various cellular events, both in physiological and pathological conditions. CircRNAs work as competing endogenous RNAs, thus sponging miRNAs and RNA-binding proteins and competing with linear splicing; they have been found localized in nuclear and cytoplasmic compartments, and growing evidence has demonstrated that they can be also transported between cells by EVs [47].

The identification of new forms of RNAs has led to a scientific fervor aimed at understanding their mechanisms of action, while the discovery of circulating non-coding RNA, associated or not to EVs, emerged from high throughput RNA sequencing studies. To sort through this complex amount of information, the Extracellular RNA Communication Consortium (ERCC) developed an exRNA Atlas (https://exrna-atlas.org), collecting data from different human biofluids. This represents a map of cell–cell communication mediated by extracellular RNA, associated with vesicular and non-vesicular (RNP or lipoprotein) extracellular RNA carriers [48]. Mainly focused on non-coding RNA carried by EVs is the exoRBase (http://www.exoRBase.org), which collects circRNA, lncRNA, and mRNA data derived from RNA-seq data analyses of human blood exosomes [49]. Conversely, focused on miRNAs is the EVmiRNA (http://bioinfo.life.hust.edu.cn/EVmiRNA#!/) database, which collects comprehensive miRNA profiles in EVs [50].

Despite the technical constraints of each study, with limits and biases associated with RNA extraction and/or vesicle isolation protocols, it is, however, possible to make some general considerations about the most abundant types and the roles of non-coding RNAs in the EVs. Starting from the pioneering studies of Valadi [25] and Nolte-′t Hoen and colleagues [51], subsequent research has confirmed that EVs are enriched in small non-coding RNAs, such as vault RNA, Y-RNA, and specific tRNAs [51], repeat and transposable elements, small nuclear RNA (snRNA), and signal recognition particle RNA (srpRNA) [52].

Increasing evidence has suggested that the presence of these ncRNAs in the EVs is a highly controlled and specific process; therefore, the evaluation of the mechanisms underlying the sorting of these ncRNAs in the EVs is among the most interesting questions for researchers. In a study by Villarroya-Beltri et al., it was found that the most abundant miRNAs in EVs from human primary T-cells possess a specific sequence called EXOmotif (GGAG); this sequence is bound by the Heterogeneous Nuclear Ribonucleoprotein A2/B1 (hnRNPA2B1), which guides the sorting process of miRNAs into EVs [29]. In accordance with this groundbreaking study, another group demonstrated that the loading of specific miRNAs in hepatocyte-derived EVs depends on the presence of the EXO motif, GGCU, which is recognized by the RBP SYNCRIP [30]. Overall, these findings provide the basis for the identification of further sequence-specific transport systems.

Another important aspect analyzed by the scientific community concerns the abundance of ncRNAs in EVs as compared with the EV-producing cells. Today, RNA-seq technologies allow the qualitative comparison of the RNA species in the two biological samples; however, different limits still exist for the definition of the relative abundances. It remains difficult to assess whether an enrichment in the EVs exists. The absence of exclusive housekeeping genes for vesicle samples [53], as well as the heterogeneity of EVs produced by the same cell type [28], still represent important challenges to be addressed.

## 3. EV-Associated ncRNAs Contribute to Tumor Pathobiology

The generation of a complex network of interactions among different cells of the bone marrow microenvironment is critical in MM pathophysiology. In this context, EVs released by several cell types, including MM cells and host cells, contribute to MM onset and progression [54,55]. In this section, we will discuss findings highlighting the pivotal contribution of EV-ncRNAs to MM onset and progression, focusing on different aspects of MM pathobiology.

### 3.1. EV-ncRNAs in MM Proliferation and Spreading

The EV-mediated crosstalk between MM cells and bone marrow (BM) cells has been found to affect the proliferation, survival, and aggressiveness of cancer cells [55,56]. The ncRNA content of EVs is accountable for this phenomenon and seems to involve EVs from both cancer and normal cells [57].

Cheng and colleagues observed that three different human MM cell lines release EVs with a high content of *miR-21* [58], which is involved in MM initiation and recurrence [59]. In this pioneering study, the authors found that EVs from the OPM2 cell line were able to promote MSC proliferation and cancer-associated fibroblast (CAF) transformation by increasing *SDF-1*, *FAP*, and *α-SMA* expression levels. The observed effects were ascribed to the presence of *miR-21* in EVs; in fact, the inhibition of *miR-21* led to a decrease in the CAF markers [58]. Along with *miR-21*, the authors also examined the role of *miR-146a*, since it was also found to be highly abundant in EVs and it was correlated with cancer progression [60]. They demonstrated that this miRNA can be transferred to MSCs after treatment with OPM2 EVs, inducing an increase in IL-6, although the underlying mechanism remains to be clarified [58]. Other studies correlated the presence of *miR-146a* in MM EVs with their pro-inflammatory effects in target cells. De Veirman and colleagues correlated this to the IL-6 increase in MSCs treated with EVs from U266 [61]. In the same study, the authors demonstrated that EV *miR-146a* stimulated in MSCs the expression and the release of factors affecting MM viability and migration, namely CXCL1, IP-10, and CCL5 [61]. The authors hypothesized the involvement of the Notch signaling pathway in such effects. Indeed, Notch signaling inhibition using the gamma secretase inhibitor DAPT resulted in a reduced expression of its target genes, *Hes 5* and *Hey 2*, and of the pro-inflammatory cytokines released by MSCs transfectd with *miR-146* mimics [61].

Several data revealed that also EVs from bone marrow stromal cells (BMSCs) of MM patients play a key role in disease progression. A recent study showed that the EVs released by BMSCs isolated from MM patients have a different miRNA profile compared to EVs from healthy donors. In particular, the authors demonstrated that MM-BMSCs EVs carry higher amounts of *miR-10a*, *miR-346*, and *miR-135b*. Among these miRNAs, *miR-10a* was transferred to MM cells by MM-BMSCs EVs, leading to tumor cell proliferation. Bioinformatic analysis identified TAK1 and βTRC as being positively regulated by *miR-10a*; interestingly, by using a β-TRC inhibitor, *miR-10a*-mediated MM cell proliferation was arrested [62].

Roccaro et al. treated MM cells with BMSC-derived EVs from healthy donors (HD) or MM patients; the authors observed a reduction in the proliferation of MM cells when treated with HD-BMSCs EVs. They further analyzed the miRNA content of EVs and found a higher level of *miR-15a* in HD-BMSC-derived EVs compared to those of MM and MGUS-BMSCs [56]. Moreover, *miR-15a*, which is considered a tumor suppressor miRNA in MM [63], was underexpressed in MM cells. To better understand the role of *miR-15a* in HD-BMSCs’ EV-mediated anti-proliferative effect on MM cells, the authors isolated BMSCs’ EVs from wild-type or *miR-15a/16-1^−/−^* mice and found that *miR-15a/16-1^−/−^* BMSC-derived EVs did not stimulate MM cell proliferation. By performing gain and loss of function assays, the authors confirmed that *miR-15a* acts as a tumor suppressor miR [56].

The involvement of long non-coding RNAs cargo of MSC EVs in the promotion of MM cell proliferation has been also documented. A recent study revealed that *LINC00461* is highly expressed both in MM cell lines and in MM patients’ plasma cells, where it exerts an anti-apoptotic role [64]. *LINC00461* is transferred to MM cells through MSC-derived EVs and it sponges *miR-15a* and *miR-16*, inhibiting their expression and upregulating their target, Bcl2, which in turn favors MM cell proliferation and suppresses apoptosis [64].

### 3.2. EV-ncRNAs in MM Angiogenesis

Bone marrow angiogenesis is essential for MM progression. Recent studies have shown that MM EVs enhance angiogenesis since they increase the viability of the endothelium by modulating different signaling pathways, such as the STAT3, JNK, AKT, p38, and p53 pathways [65]. MM EVs have been shown to induce endothelial cell proliferation [66] by delivering several angiogenic factors such as angiogenin, basic fibroblast growth factor (bFGF), and vascular endothelial growth factor (VEGF) [65] and by inducing endothelial cells to secrete IL-6 and VEGF [67].

ncRNAs have been also demonstrated to act as molecular mediators of EV-mediated angiogenesis.

Interestingly, a study demonstrated that MM EVs released under hypoxic conditions (H-EVs) significantly increase the tube formation of endothelial cells compared with EVs derived from MM cells under normoxic conditions (N-EVs) [68]. By analyzing the miRNA profile of EVs, the authors found that H-EVs’ miRNA content differed from that of N-EVs; in particular, H-EVs had higher levels of *miR-210* and *miR-135b* compared to N-EVs. Nevertheless, *miR-210* was upregulated in the endothelial cell line HUVEC under hypoxic conditions, regardless of treatment with MM EVs; *miR-135b* level, instead, was dependent on EV treatment. H-EVs’ *miR-135b* was transferred to endothelial cells and increased angiogenesis both in vitro and in vivo through the suppression of the inhibitor of hypoxia-induced factor 1 (FIH-1), which is a negative regulator of HIF-1 [68].

Among ncRNAs playing a key role in MM angiogenesis, there is *piRNA-823*. It is noteworthy that MM patients’ plasma cells have a higher level of *piRNA-823* compared to healthy individuals’ cells, and overexpression of this piRNA stimulated MM cell proliferation and angiogenesis [69]. Recently, it was found that MM cells package *piRNA-823* in their EVs for subsequent transfer to endothelial cells, enhancing their proliferation through the inhibition of pro-apoptotic proteins and triggering of reactive oxigen species (ROS); the mechanisms underlying these effects have not been clarified yet. Moreover, EV *piRNA-823* could promote endothelial cell release of IL-6 and VEGF, thus favoring angiogenesis. Finally, *piRNA-823* triggered the expression of ICAM-1 and CXCR4, two essential molecules in the cell invasion process [70].

### 3.3. EV-ncRNAs in MM Bone Disease

The main complication of MM is osteolytic bone disease, which affects more than 80% of patients [71,72,73]. In physiological conditions, bone homeostasis is maintained by the balanced activity of osteoblasts (OBs) and osteoclasts (OCs). In MM, such a balance is lost since osteoblast functions are inhibited while osteoclasts are hyper-activated, thus promoting the occurrence of osteolytic lesions [72,73,74]. This condition is due to the crosstalk within the bone marrow niche, among MM cells and surrounding cells, mediated by secreted factors, including EVs [71,74].

In recent years, increasing evidence has shown that EVs, and specifically their ncRNA content, are responsible for the onset of bone disease since they can promote osteoclast activation and inhibit the osteogenic differentiation of mesenchymal stem cells (MSCs) [75,76,77,78,79].

A groundbreaking study by Li and colleagues has firstly demonstrated that MSCs internalize MM cell-derived exosomes, which causes a reduction in MSC bone nodule formation, a key marker of osteogenic differentiation, compared to the control cells that were treated with phosphate-buffered saline (PBS) or with MM-conditioned medium depleted of exosomes [77]. They identified in MM-EVs the *lncRUNX2-AS1*, a long non-coding RNA that is negatively correlated with the expression of RUNX2, a marker of osteogenic differentiation [72]. EV-carried *lncRUNX2-AS1* forms a duplex with *RUNX2* pre-mRNA, thus blocking its splicing and leading to the inhibition of the osteogenic differentiation of target cells. The in vitro results were confirmed also in in vivo experiments by treating mice xenografted with MM cells with GW4869, an inhibitor of EV secretion. The MSCs derived from mice treated with GW4869 presented higher levels of *RUNX2* and lower *lncRUNX2-AS1* expression with respect to MSCs derived from mice of the control group [77].

Moreover, another group confirmed that treatment of MSCs with MM-EVs inhibited osteoblast differentiation and reported the presence of some miRNAs in EVs with a putative role in MM bone disease [78]. They found that the MSC treatment with MM-EVs caused an increase in *miR-103a-3p* levels, which inhibited bone formation through *RUNX2* targeting [80]. Indeed, *miR-103a-3p* upregulation in MSCs led to decreased osteoblastogenesis [78].

Similarly, in a recent study, we identified a panel of miRNAs packed in MM-EVs from MM cell lines or primary plasma cells from bone marrow aspirates that can negatively regulate osteogenesis [79]. Among them, we identified and characterized *miR-129-5p*, which targets various mRNAs involved in osteoblast differentiation [81,82,83,84]. Moreover, we observed that this miRNA is transferred to MSCs by MM-EVs, causing a downregulation of *SP-1*, a transcription factor implicated in osteogenesis through the targeting of alkaline phosphatase (*ALPL*) [79] and also involved in MM cell proliferation [85].

**Figure 1:**
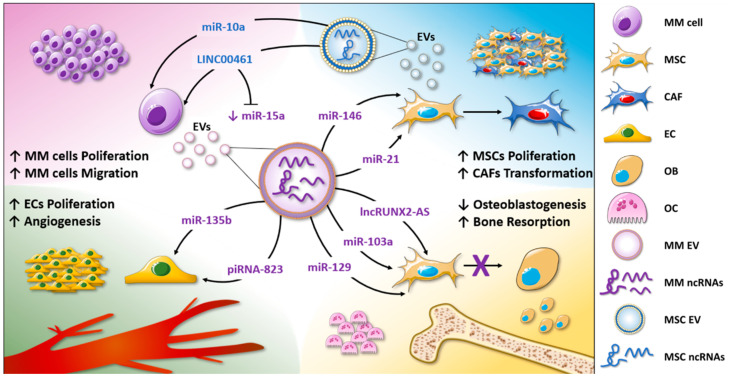
EV-associated ncRNAs are involved in MM biological functions. The non-coding RNA content of MM-EVs is involved in the enhanced tumor cell proliferation and migration (purple corner), in MSC proliferation, and CAF transformation (light-blue corner); additionally, by delivering ncRNAs, MM-EVs induce angiogenesis, promoting endothelial cell proliferation and the release of angiogenic factors (green corner). Finally, EVs regulate MM bone disease by altering osteoclast and osteoblast functions (yellow corner).

Overall, in this section, we have reported the studies that correlate the ncRNA content of EVs with disease onset or progression. The evidence discussed above is summarized in the Figure 1 cartoon.

## 4. EV-ncRNAs Mediate Drug Resistance in MM

Although considerable progress has been made in the development of effective treatment strategies, MM remains an incurable malignancy. Different therapeutic approaches have been adopted over time, which include immunomodulatory drugs (pomalidomide), proteasome inhibitors (bortezomib, carfilzomib and, ixazomib), histone deacetylase inhibitors (panobinostat), and monoclonal antibodies (elotuzumab and daratumumab) [9,86]. One of the limitations of the existing treatment is the emergence of drug resistance [9,86].

Accumulating evidence shows that EVs could participate in this process [87,88]. It was found that BMSC-derived EVs isolated from both normal donors and MM patients could induce drug resistance in human MM cells; in fact, EVs increased MM cell viability by 25% in the presence of bortezomib and by 9% in the absence of the drug [87]. Furthermore, Faict and colleagues highlighted that MM cell treatment with melphalan or bortezomib induced the release of EVs with a higher amount of acid sphingomyelinase, which could have a role in the MM drug resistance mechanism [88].

Intriguingly, results from different studies have underlined that ncRNAs contained in EVs are partially responsible for this phenomenon [89,90]. In a study focused on bortezomib resistance, the authors identified, through a microarray approach, the miRNA content of EVs isolated from the peripheral blood of bortezomib-resistant (Bz-resistant) MM patients. Firstly, the authors found a significant difference in the total RNA content of EVs purified from Bz-responsive and Bz-resistant groups. In particular, a higher concentration of EV-RNA was found in Bz-resistant samples; the rationale underlying this has yet to be investigated. Furthermore, 83 miRNAs were found to be more abundant and 88 less abundant in the EVs of Bz-resistant patients compared to EVs from Bz-responders. miRNAs with a different amount included *miR-513a-5p*, *miR-20b-3p*, and *let-7d-3p* (higher amount) and *miR-125b-5p*, *miR-19a-3p*, *miR-21-5p*, *miR-20a-5p*, *miR-17-5p*, *miR-15a-5p*, and *miR-16-5p* (lower amount). By performing computational analyses, the authors speculated that these miRNAs could have a role in the bortezomib-resistance mechanism as they participate in post-transcriptional regulation by modulating co-factors of the MAP kinase and ubiquitin-conjugating enzyme activity pathways [89].

Along with cancer cells, BMSCs may take part in the drug resistance process. BM stromal cells are involved both in the beginning and in the maintenance of the drug-resistant phenotype of MM cells [91,92,93].

Xu and colleagues have studied the role of EV-ncRNAs transferred from MSCs to MM cells in the resistance to proteasome inhibitors [90]. The authors demonstrated that treatment of MM cells with EVs from MSCs isolated from bortezomib-resistant patients (r-MSCs) reduced cancer cell sensitivity to proteasome inhibitors; conversely, treating MM cells with EVs from MSCs of bortezomib-sensitive patients (s-MSCs) did not affect the tumor response to therapy. By analyzing the EV content, the authors identified the presence of *PSMA3* and *PSMA3-AS1* transcripts in MSC-derived EVs, especially in vesicles isolated from resistant patients’ cells. PSMA3 encodes the proteasome type-3 alpha subunit [94], whereas *PSMA3-AS1* is a lncRNA that modulates PSMA3 levels by increasing its stability. *PSMA3* and *PSMA3-AS1* expression levels were upregulated in MM cells treated with r-MSC-EVs but not in cells treated with s-MSC-EVs. The upregulation of these two transcripts led to enhanced proteasome activity, which could explain the resistance to proteasome inhibitors. These results were confirmed also in vivo using U266-luc mice. The authors demonstrated that *PSMA3-AS1* downregulation via siRNAs increased the sensitivity of MM cells xenografted in mice to proteasome inhibitors [90].

## 5. EV-ncRNAs as Diagnostic and Prognostic Biomarkers in MM

Nucleic acids circulating in biological fluids represent an important source of biomarkers for the early detection of cancer and the monitoring of treatments. This preventive, diagnostic, and monitoring approach is currently known as liquid biopsy, a minimally invasive procedure for the patient. In particular, the advantage of liquid biopsy in oncology is to obtain the tumor molecular profile when the biopsy from the primary or metastatic tumor is not feasible due to risks associated with the surgical procedure.

The existence of extracellular RNAs [95,96], also called circulating tumor RNAs (ctRNAs), in serum or plasma as well as in other biological fluids such as urine, saliva, cerebrospinal, seminal, and ascitic fluid, has highlighted the possibility that they may represent ideal candidates as tumor biomarkers.

To date, increasing scientific evidence has indicated that several miRNAs and lncRNAs are found in the plasma of MM patients and that these may correlate with the disease stage, thus representing potential biomarkers.

In this context, the majority of studies have focused on circulating miRNAs differentially expressed in the distinctive monoclonal gammopathies. In a study by Jones et al., differences in serum miRNA levels of healthy subjects and MM and MGUS patients were identified. Among these, *miR-720* was higher in MM and MGUS patients compared with healthy subjects, while *miR-1308* was lower; also, the combination of *miR-1246* and *miR-1308* levels might be used to discriminate MGUS from MM [97]. Similarly, plasma levels of *miR-92a* were found to be lower in MM patients than in healthy and MGUS individuals [98]. Interestingly, increased levels of *miR-214* and *miR-135b* were found in the serum of MM patients with osteolytic lesions, thus representing a predictive marker of MM bone disease [99].

More recently, a study characterized the peripheral blood plasma transcriptomic profile of newly diagnosed and relapsed and refractory MM patients, and this was compared to that of healthy individuals [100]. The authors not only identified differences in the expression of protein-coding genes but also reported variations in the levels of some non-coding genes; these include antisense genes such as *FAM83C-AS1*, *ZNF32-AS1*, *TMC3-AS1*, and *TAT-AS1*, long intergenic noncoding RNA (LincRNA) such as *LINC00863*, *LINC01123*, *LINC00349*, *LINC00677*, and *LINC00462*, and microRNAs including *miR-301A*, *miR-378H*, *miR-425*, and *miR-647* [100].

Circulating non-coding RNA has proven to be a predictive tool to monitor patient response to therapies with lenalidomide and dexamethasone [101]. In this study, the authors analyzed the profiles of miRNAs from the serum of patients with relapsed/refractory MM (RRMM) exhibiting different responses to treatment. Although various miRNAs were differentially expressed between the two groups, the levels of five of these (*miR-26a-5p*, *miR-29c-3p*, *miR-30b-5p*, *miR-30c-5p*, and *miR-331-3p*) were significantly reduced in partially-responsive patients [101].

To date, the major limitations for the use of ctRNAs as biomarkers in clinical settings are their instability in biological fluids and the difficulty of processing and analysis [102,103].

However, RNAs are also contained in EVs that, thanks to their stability and abundance, may represent today a great opportunity for cancer biomarker development, also for MM.

In particular, increasing evidence indicates that the analysis of ncRNA levels in serum/plasma EVs could be a useful tool for the differential diagnosis of patients with different gammopathies [104] and the prediction of patient outcomes [105]. For example, a comparison of the miRNAs in the vesicles of patients with MM and SMM and healthy subjects showed differences between the different groups; moreover, in the same study, it was found that the levels of miRNAs associated with vesicles are different from those circulating in serum [104]. Among miRNAs differentially packed in EVs of affected subjects, *let-7c-5p*, *miR-20a-5p*, *miR-103a-3p*, *miR-140-3p*, and *miR-185-5p* were consistently reduced in patients with MM than in those with SMM, while *miR-4505* and *miR-4741* were higher [101].

In our recent study, we found higher levels of *miR-129-5p* in vesicles isolated from the bone marrow of MM patients than in SMM [79]; since this miRNA targets different mRNAs involved in osteoblast differentiation [81,82,83,84], its presence in plasma EVs could be relevant to discriminate between the two pathological conditions.

In addition to mRNAs, lncRNAs delivered by EVs have the potential to distinguish patients with different monoclonal gammopathies. The description of the lncRNA content of vesicles isolated from the peripheral blood of MM, MGUS, and healthy individuals showed differential amounts of various lncRNAs [106]; in particular, among 84 identified lncRNAs, the levels of *PRINS* (psoriasis susceptibility-related RNA gene induced by stress) discriminated patients with monoclonal gammopathies from healthy subjects. Importantly, the abundance of *PRINS* in the EVs from patients correlated with clinical parameters such as bone marrow plasma cell infiltration rate, albumin, creatinine, and lactate dehydrogenase levels [106].

The identification of EV-associated ncRNAs may have a prognostic impact. In a cohort of MM patients, the correlation between EV-miRNAs and patient outcomes revealed that the levels of *let-7b* and *miR-18a* were predictors of overall survival; in particular, low levels of these two miRNAs were associated with poor outcomes [105].

EV-associated ncRNAs have also emerged as tools for the non-invasive evaluation of the therapeutic response. For example, the identification of the miRNA profile of EVs from the MM patients’ plasma revealed different miRNA patterns between patients who were either responsive or resistant to bortezomib treatment. Among them, levels of *miR-16-5p*, *miR-15a-5p*, *miR-20a-5p*, and *miR-17-5p* were reduced in the EVs from resistant patients [89].

**Figure 2:**
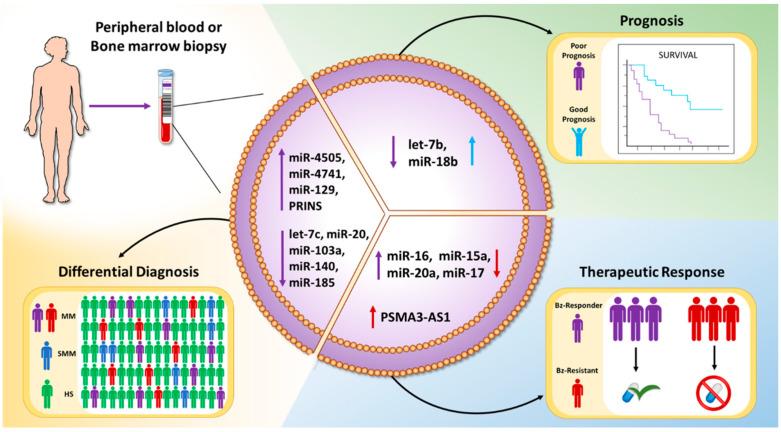
Schematic representation of the clinical value of EV-associated ncRNAs: EVs can be isolated from the peripheral blood or the bone marrow aspirates of patients with different gammopathies. EV-ncRNAs may serve as biomarkers for the differential diagnosis, prognosis, or prediction of therapeutic response.

Altogether, in this section, we have reported findings suggesting that EV-associated ncRNAs represent new biomarkers for the differential diagnosis of monoclonal gammopathies and the monitoring of the therapeutic response (Figure 2).

## 6. Conclusions

In summary, we have reported and discussed evidence indicating that ncRNAs contribute to the pathogenetic activity of EVs in MM. In particular, the involvement of ncRNAs contained in EVs in the proliferation of tumor and microenvironmental cells, drug resistance, increased angiogenesis, and bone disease associated with MM is nowadays clear.

However, it has to be underlined that, although many works confirmed a lower abundance of miRNAs in EVs respect to other ncRNAs [107,108,109], most of the functional studies about the EV-mediated transfer of non-coding RNAs, some of which are reviewed above, focus on miRNAs. Further in-depth characterization of the ncRNA cargo of vesicles is therefore necessary and will allow us to identify new possible therapeutic targets as well as to develop diagnostic and prognostic tools. Furthermore, additional validation studies of the identified EV-ncRNAs are needed before translation into the clinical setting.

The tables reported below summarize major findings on the biological (Table 1) and clinical (Table 2) impact of EV-ncRNAs in MM.

## Figures and Tables

**Table 1:** Effects of EV-ncRNAs on MM pathobiology.

EV Source	EV-ncRNA Species	Target Cells	Biological Function	Reference
**Tumor Proliferation**				
MM cells	*miR-21*	MSCs	MSC proliferation; increase in SDF-1, FAP, and α-SMA expression levels; CAF transformation.	[58]
MM cells	*miR-146*	MSCs	MSC proliferation; IL6, CXCL1, IP-10, and CCL5 release through Notch signaling.	[58,61]
BMSCs from MM patients	*miR-10a*, *miR-346*, *miR-135b*	MM cells	*miR-10a* transfer leads to MM cell proliferation.	[62]
BMSCs from healthy donors and MM patients	*miR-15a*	MM cells	*miR-15a* is enriched in EVs from healthy donors and exerts anti-proliferative functions in MM.	[56]
MSCs	*LINC00461*	MM cells	Inhibition of *miR-15a* and *miR-16*-mediated BCL2 reduction; MM cell proliferation.	[64]
**Tumor Angiogenesis**				
MM cells under hypoxic condition	*miR-135b*	Endothelial cells	In vitro and in vivo increase in angiogenesis by FIH-1 inhibition.	[68]
MM cells	*piRNA-823*	Endothelial cells	Enhanced endothelial cell proliferation by apoptotic proteins and ROS production inhibition; increase in IL6, VEGF, ICAM-1, and CXCR4.	[70]
**Bone Disease**				
MM cells	*lncRUNX2-AS1*	MSCs	Inhibition of MSC osteogenic differentiation by RUNX2 reduction.	[77]
MM cells	*miR-103a-3p*	BM-MSCs	Inhibition of MSC osteogenic differentiation.	[78]
MM cells and MM BM plasma	*miR-129-5p*	MSCs	Inhibition of MSC osteogenic differentiation by ALPL reduction.	[79]

**Table 2:** Diagnostic and prognostic values of EV-ncRNAs.

EV Source	EV-ncRNA Species	Diagnostic/Prognostic Finding	Number of Patients Enrolled	Reference
Bz-resistant and Bz-responder MM patients	*miR-16-5p*, *miR-15a-5p*, *miR-20a-5p*, *miR-17-5p*	miRNAs reduced in the EVs from patients resistant to the treatment	Bz-resistant: 3 Bz-responder: 3	[89]
MSCs from Bz-resistant MM patients	*PSMA3-AS1*	Enhanced proteasome activity leading to resistance to proteasome inhibitors.	Bz-resistant: 45 Bz-sensitive: 12	[90]
SMM, MM patients, and healthy donors	*let-7c-5p*, *miR-20a-5p*, *miR-103a-3p*, *miR-140-3p*, *miR-185-5p*, *miR-4505*, *miR-4741*	*let-7c-5p*, *miR-20a-5p*, *miR-103a-3p*, *miR-140-3p*, and *miR-185-5p* levels were reduced, while *miR-4505* and *miR-4741* were increased, in MM with respect to SMM patients.	Healthy individuals: 16 SMM patients: 20 MM patients: 20	[104]
MM patients	*let-7b* and *miR-18a*	*let-7b* and *miR-18a* low levels were associated with reduced OS.	MM patients: 156	[105]
MGUS, MM patients, and healthy donors	*PRINS*	*PRINS* levels discriminate patients with monoclonal gammopathies from healthy subjects.	Healthy donors: 30 MGUS patients: 49 MM patients: 50	[106]

## Abbreviations

EVs	Extracellular vesicles
MM	Multiple myeloma
MGUS	Monoclonal gammopathy of undetermined significance
SMM	Smoldering multiple myeloma
BM	Bone marrow
BMSCc	Bone marrow stromal cells
ncRNAs	Non-coding RNAs
miRNA	MicroRNAs
lncRNA	Long non-coding RNA
lincRNA	Long intergenic non-coding RNAs
piRNA	Piwi-interacting RNA
MSC	Mesenchymal stem cells
